# Improving Deep Drawing Quality of DD13 Sheet Metal: Optimization of Process Parameters Using Box–Behnken Design

**DOI:** 10.3390/ma18071424

**Published:** 2025-03-23

**Authors:** Ilhan Celik, Abdullah Tahir Şensoy, Gokhan Seven, Dilek Cicek

**Affiliations:** 1 Department of Mechanical Engineering, Faculty of Engineering and Natural Sciences, Samsun University, Samsun 55420, Türkiye; ilhan.celik@samsun.edu.tr; 2 Faculty of Mechanical Engineering, Delft University of Technology (TU Delft), Mekelweg 2, 2628 CD Delft , The Netherlands; 3 Department of Biomedical Engineering, Faculty of Engineering and Natural Sciences, Samsun University, Samsun 55420, Türkiye; 4 Department of Sheet Metal Production, Sampa Automotive Industry and Trade Inc., Samsun 55300, Türkiye; gokhanseven_5534@hotmail.com; 5 Department of Mechanical Engineering, Institute of Graduate Programs, Samsun University, Samsun 55420, Türkiye

**Keywords:** Particle Swarm Optimization, Box–Behnken design, deep drawing, response surface method

## Abstract

This study presents a comprehensive analysis of the earing and thinning ratios in the deep drawing process of DD13-grade sheet metal. The variable parameters investigated include press descent speed (x_1_), blank holder pressure (x_2_) and punch pressure (x_3_). These parameters have been carefully selected based on production experience and preliminary experiments, and appropriate ranges of values have been established. A distinctive feature of this research is its focus on specimens with a deep drawing ratio greater than 2. The relationships between the input parameters and the response parameters were determined using the Box–Behnken design (BBD). Analysis of variance (ANOVA) for the regression models developed for earing and thinning ratios gave R^2^ values of 0.98 and 0.97, respectively, indicating robust predictive ability of the models. For the earing ratio, the optimal values of x_1_, x_2_ and x_3_ were found to be 27.38, 10 and 22, respectively. When evaluated using the same algorithm for uniform wall thickness, the optimum values were found to be 26.55, 10 and 22, respectively. The final earing and thinning ratios for all experimental runs were 3.79 and 21.19, respectively. The results of this study show that by optimizing certain parameters in the deep drawing process, the disadvantages associated with this manufacturing method can be significantly reduced.

## 1. Introduction

Research conducted to enhance the usability of various materials used in industry and optimize their performance significantly contributes to the development of industrial processes. The DD13 steel examined in this study is a material utilized in various industrial applications and has a wide range of uses in the engineering field. Deep drawing processes refer to the shaping of metal sheets by pulling them into a specific mold and are a commonly used metal-forming method in industrial production. During this process, the metal sheet material undergoes plastic deformation within the mold and takes on the desired final product shape. Deep drawing processes have diverse applications across many sectors, from the automotive industry to household appliances. In this context, the optimization of deep drawing processes represents an important area of research. Optimizing deep drawing processes can increase production efficiency, make more effective use of material resources, and improve the quality of the final product. This optimization process is achieved by carefully adjusting various parameters (e.g., press force, material thickness, press speed). Notably, Box–Behnken design (BBD) is a widely used statistical design method for understanding and optimizing complex relationships among various parameters [1,2]. This methodology aims to create mathematical models to assess the effects of interacting factors while minimizing the number of experimental studies. In this way, it contributes to the faster and more effective execution of production processes [3].

Researchers have extensively investigated optimization methods to enhance the efficiency, quality, and cost-effectiveness of metal-forming processes. Studies on the optimization of metal-forming processes consider factors such as energy consumption, shape optimization, and the impact of process parameters on shaping quality and energy efficiency [4,5,6]. Other studies examining the use of finite element simulations and optimization strategies in forging processes emphasize the importance of accurate modeling and systematic optimization to achieve desired outcomes in metal forming operations [7,8]. Innovative approaches, such as reliability-based economic optimization and sensitivity analysis methods, are available for optimizing sheet metal-forming processes under uncertainty [9,10]. Furthermore, advancements in optimization techniques focusing on structural and process parameters in sheet metal-forming operations indicate a shift towards a more holistic optimization approach that considers both design and process parameters [11,12]. In optimizing the earing ratio and uniform thickness in the deep drawing process, various methodologies and parameters play a crucial role. The Taguchi method has proven effective in determining optimal parameters such as material thickness and drawing depth, significantly influencing the earing phenomenon during deep drawing [13]. Additionally, the geometry of tools used in the process has been shown to affect formability and thickness distribution, with numerical analyses aligning closely with experimental results [14]. Moreover, the introduction of advanced techniques like hydroforming can enhance the forming limits of materials, thereby improving thickness uniformity and reducing defects such as thinning and earing [15]. The optimization of process parameters, including blank holder force and die radius, is essential for achieving uniform thickness and minimizing defects [16,17]. Furthermore, studies indicate that the use of innovative approaches, such as variable blank holder force paths, can effectively reduce thickness variation during the deep drawing process [18]. A comprehensive understanding of the interplay between process parameters and material properties is vital for optimizing the deep drawing process to achieve desired earing ratios and uniform thickness. The relationship between metal forming and optimization has the potential to guide future studies aimed at developing more sustainable and competitive production processes in engineering applications.

To the best of the authors’ knowledge, this is the first study in the literature to apply response surface methodology to samples with a deep drawing ratio greater than 2. Therefore, this study presents a new perspective for conducting press operations on steel sheet materials more specifically and effectively. In this research, which employs the Box–Behnken design (BBD) method, effective process parameters and their operating ranges have been identified, and regression models have been established between the input parameters and the response parameters. The regression equations formulated separately for the response parameters, namely earing and thinning ratios, have been designated as objective functions for the optimization process. This approach allowed for the calculation of the optimal values of process parameters that minimize the objective functions.

## 2. Experimental Method

### 2.1. Experimental Material

In this study, DD13-grade steel sheets were used as the experimental material. Circular blanks with a diameter of 430 mm were obtained from a roll with a thickness of 3 mm and a width of 436 mm, using a blanking die with an outer cutting diameter of 430 mm. The chemical composition of the samples is provided in Table 1.

### 2.2. Experimental Set-Up

**Figure 1:**
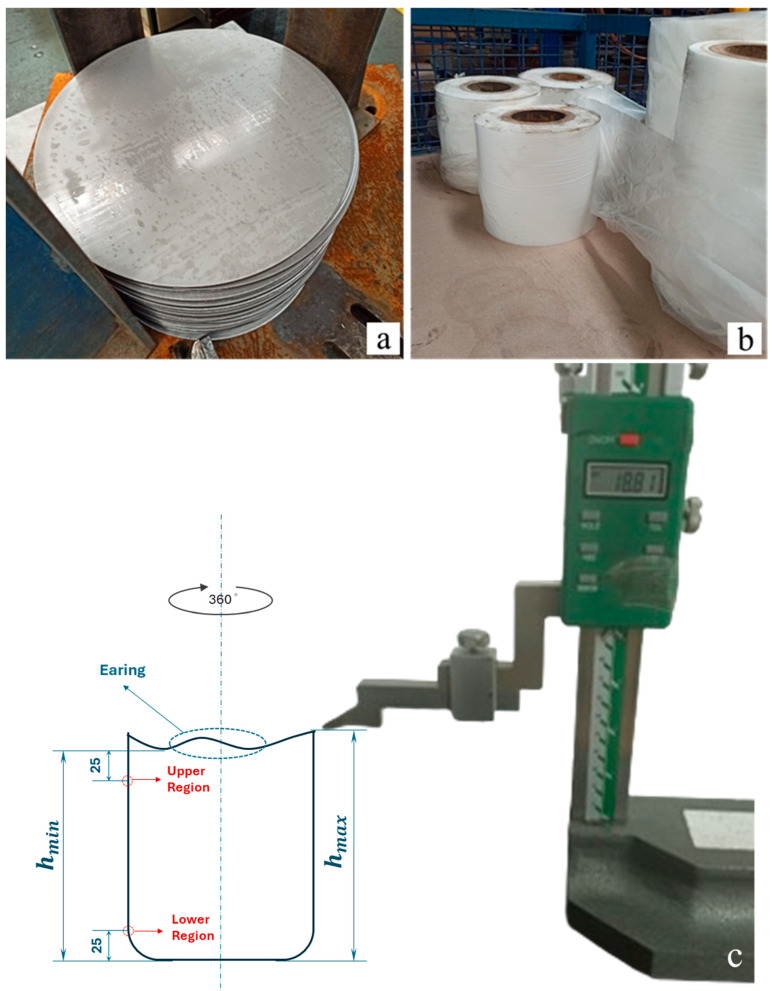
(**a**) Circular blank with a diameter of 430 mm, (**b**) nylon used in the deep drawing operation, (**c**) schematic representation of dimensional measurements and geometric analysis of deep-drawn products.

In this study, experiments were conducted using a hydraulic press [19] (Yucel Makina Hydraulic Press Man. Ind. Trade. Co., Ltd., Istanbul, Türkiye) with a punch pressure of 25 MPa, located within SAMPA Automotive Industry and Trade Inc. The hydraulic press, where deep drawing operations were performed, allows for the press descent speed to be adjusted as desired for the deep drawing process. The press descent speed was expressed in percentage units (%) due to the operating principle of the hydraulic system. The system defines the maximum speed as 100%, allowing the operator to select the appropriate speed for the deep drawing process based on the material properties. In line with this technical requirement, the press descent speed in this study has been expressed in percentage (%) units. The deep drawing mold used in the study consists of a male part with a diameter of 218.5 mm and a height of 280 mm, and a female part with a diameter of 220 mm. As shown in Figure 1a, circular blanks with a diameter of 430 mm were used in the study. To reduce friction between the blank and the die, a nylon material with a thickness of 1.8 microns was employed (Figure 1b). This material serves both as a lubricant and prevents the formation of scratches on the material surface. In the study, the circular blank was placed on the blank holder, and deep drawing operations were carried out by adjusting values such as the designated press stroke speed, punch pressure, and blank holder pressure.

To determine the maximum and minimum height of a deep-drawn product, the process begins with a visual inspection to identify potential high points, followed by quantitative measurement using the gauge shown in Figure 1c. This process involves measuring the vertical distances on the side surfaces of the cylindrical sample to determine the amount of earing. The measurement part of the gauge is brought into contact with the highest point of the product. The product is then rotated 360 degrees to check if there is a point higher than the one identified. This way, the maximum height (h_max_) is measured. On the other hand, to determine the minimum height (h_min_), the measurement point of the gauge is touched to the shortest point of the product (the area where elongation is visually the least). The product is again rotated 360 degrees to find the lowest point.

### 2.3. Box–Behnken Design (BBD)

The experiments designed with the parameters and levels given in Table 2 resulted in a total of 15 design combinations for 3 continuous variables, determined using Box–Behnken design.

A second-degree regression model has been established between the obtained response values and the input parameters:

(1) $$y=\beta_{0}+\sum_{i=1}^{k}\beta_{i}X_{i}+\sum_{i=1}^{k}\beta_{ii}X_{i}^{2}+\sum_{i=1}^{k-1}\sum_{j=i+1}^{k}\beta_{ij}X_{i}X_{j}+\varepsilon$$

In the equation, $\beta_{0}$ represents the constant coefficient, $\beta_{i}$ represents the coefficients of the linear terms of the regression model, $\beta_{ii}$ represents the coefficients of the quadratic terms, $\beta_{ij}$ represents the coefficients of the interaction terms, $X_{i}$ denotes the specified independent variables (factors of the experimental design), and ε represents the error term. According to Equation (1), the regression model established for the first response parameter, the earing ratio, using the MINITAB (Minitab 22.0, Minitab Inc., State College, PA, USA) statistical software is presented in Equation (2):

(2) $$y_{1}=18.5-0.493x_{1}+0.1951x_{2}-0.108x_{3}+0.03849x_{1}^{2}+0.000423x_{2}^{2}+0.000950x_{3}^{2}-0.00705x_{1}x_{3}+0.001524x_{2}x_{3}$$

According to the analysis of variance (ANOVA) results, the R^2^ and Adj-R^2^ values for the model in Equation (2) were calculated to be 0.987 and 0.962, respectively. Statistical indicators demonstrate that the model’s predictive capability is very high.

Following the same method, the regression model established for the second response parameter, the thinning ratio, is presented in Equation (3):

(3) $$y_{2}=-39.2-0.23x_{1}+2.350x_{2}-0.227x_{3}+0.0641x_{1}^{2}-0.00191x_{2}^{2}+0.00264x_{3}^{2}-0.01406x_{1}x_{2}-0.00852x_{1}x_{3}-0.008422x_{2}x_{3}$$

According to the analysis of variance (ANOVA) results, the R^2^ and Adj-R^2^ values for the model in Equation (3) were calculated to be 0.970 and 0.904, respectively. Statistical indicators demonstrate that the model’s predictive capability is quite strong.

### 2.4. Particle Swarm Optimization

Particle Swarm Optimization (PSO) is an optimization technique developed by James Kennedy and Russell Eberhart in 1995. It is inspired by the behavior of bird flocks or fish schools in nature. This technique utilizes a population of potential solutions, referred to as “particles”, to seek the best solution in a multidimensional search space. PSO aims to achieve optimal solutions by having each potential solution represented as a “particle” within a population. Each particle is characterized by a position and a velocity vector in the solution space.

The initial position $p_{i}(0)$ and velocity $v_{i}(0)$ of each particle are randomly selected within the defined solution space. The target function value $f(p_{i})$ at each particle’s current position is calculated. This function is defined as the objective function of the optimization problem. The best position found by each particle (denoted as ${pBest}_{i}$) and the best position of the entire swarm (denoted as gBest) are identified. In PSO, the terms pBest_i_ and gBest refer to the best solutions found during the optimization process:

pBest_i_ represents the best position (solution) that a specific particle (i) has found so far in the search space. Each particle in the swarm keeps track of its personal best solution. On the other hand, gBest represents the best position found by any particle in the entire swarm. All particles in the swarm share this information, and they are influenced by this globally best solution. These concepts help particles adjust their velocity and position, guiding the swarm toward an optimal solution.

For a minimization problem, if $f(p_{i})$ < $f({pBest}_{i}$), then ${pBest}_{i}$ is updated; if $f(p_{i})$ < f(gBest), then gBest is updated. The velocities of the particles are updated according to the following equation:

(4) $$v_{i}(t+1)=w\cdot v_{i}(t)+c_{1}\cdot r_{1}\cdot (\left.{pBest}_{i}-p_{i}(t)\right)+c_{2}\cdot r_{2}\cdot \left(gBest\left.-p_{i}(t)\right)\right.$$

Here, $v_{i}(t)$ represents the velocity of the ith particle at time t, and $p_{i}(t)$ represents the position of the ith particle at time t. The parameter w is the inertia coefficient, while $c_{1}$ and $c_{2}$ are the cognitive (individual) and social learning factors, respectively. $r_{1}$ and $r_{2}$ are random numbers between 0 and 1. The positions of the particles are updated according to the equation given in Equation (5):

(5) $$p_{i}(t+1)=p_{i}(t)+v_{i}(t+1)$$

This equation provides an iterative solution, and the particles iteratively update their positions based on the best positions achieved by any member of the swarm until either the number of iterations is reached or a predefined error criterion is met. Using the mathematical concept of the mentioned method, the optimization problem is expressed as follows:

y_min_ = f(x_1_, x_2_, x_3_) (6)

s.t.

0.28 ≥ $x_{1}$ ≥ 0.2

10 ≥ $x_{2}$ ≥ 6

22 ≥ $x_{3}$ ≥ 18

**Figure 2:**
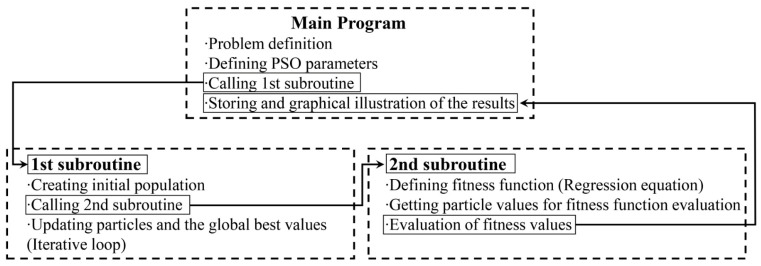
The general outline of the optimization loop coded in MATLAB [2].

Here, $x_{1},x_{2}\;and\;x_{3}$ represent the press descent speed, blank holder pressure, and punch pressure, respectively. y_min_ is the objective function dependent on these parameters. The structure of the optimization process designed using particle swarm intelligence is shown in Figure 2 [2]. To enhance computational efficiency, two subprograms have been created to run under the main program. The main program defines the optimization problem, constraints, and PSO parameters. The inertia coefficient (w), which has a significant impact on the convergence characteristics of the algorithm, is set to decrease linearly from 1.0 with a damping coefficient of 0.99. The parameters $c_{1}$ and $c_{2}$ are set to 2.05. The first subprogram initializes the swarm population, while the second subprogram calculates the fitness values (FVs). The main program tracks the best FVs found and manages the subprograms until the stopping criteria is met.

## 3. Result and Discussion

### 3.1. Results of the Box–Behnken Design

In this study, the factors in the Box–Behnken design of experiments (BBD) were determined as press descent speed, blank holder pressure and punch pressure, respectively. The earing ratios observed in the samples obtained from the experiments designed using BBD are presented in Table 3. Earing formation occurs in deep-drawn parts made from anisotropic materials. Due to the effects of planar anisotropy, the strength of the sheet material in one direction may be greater than in other directions within the plane of the sheet. As a result, even when a circular sheet is used, fluctuations at the upper edge of the deep-drawn cup, known as ears, can develop. In practical applications, these ears are trimmed to obtain the final product. Therefore, the lower the earing ratio, the less material loss will occur. 

The analysis of variance (ANOVA) for the earing ratios is provided in Table 4. According to the analysis of variance (ANOVA) results for the regression model given in Equation (2), the R^2^ and Adj-R^2^ values are calculated as 0.987 and 0.962, respectively. The statistical indicators indicate that the model’s predictive capability is very satisfactory.

**Figure 3:**
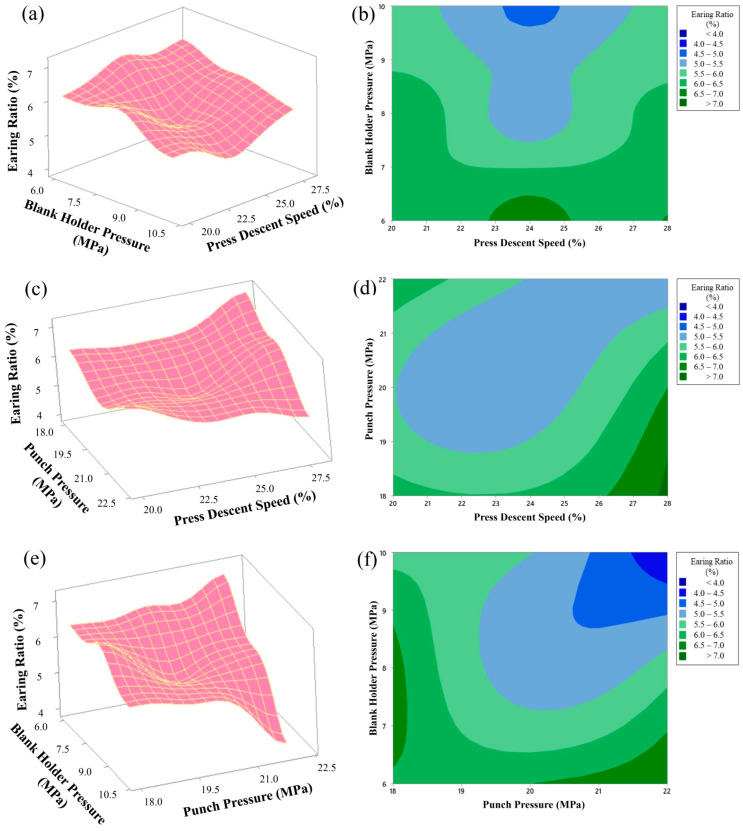
Effect of blank holder pressure, punch pressure, and press descent speed on the earing ratio: (**a**) 3D surface plot of earing ratio as a function of blank holder pressure and press descent speed, (**b**) contour plot of earing ratio as a function of blank holder pressure and press descent speed, (**c**) 3D surface plot of earing ratio as a function of punch pressure and press descent speed, (**d**) contour plot of earing ratio as a function of punch pressure and press descent speed, (**e**) 3D surface plot of earing ratio as a function of blank holder pressure and punch pressure, (**f**) contour plot of earing ratio as a function of blank holder pressure and punch pressure.

According to the response surface graphs presented in Figure 3, three main parameters affecting the earing rate in the deep drawing process were analyzed: blank holder pressure (MPa), punch pressure (MPa) and press descent speed (%). Using the Box–Behnken design, we aimed to determine the optimum values of these parameters.

In the graphs in Figure 3a,b, the effects of blank holder pressure and press descent speed variables on the earing rate were examined. Blank holder pressure was changed between 6 MPa and 10 MPa, and press descent speed was changed between 20% and 28%. The obtained results show that the earing rate decreased to minimum values (approximately 4.5–5.0%) when blank holder pressure was between 9.5 and 10 MPa and press descent speed was between 23 and 25%. This ensures that the material is shaped in a homogeneous quality.

In the graphs in Figure 3c,d, the effects of the variables punch pressure and press descent speed were examined. While punch pressure varied between 18 MPa and 22 MPa, press descent speed was again between 20% and 28%. From these graphs, an increase in the earing rate (approximately 6.5% and above) was observed when punch pressure was at low levels and press descent speed was at high levels. It was concluded that in order to obtain the optimum earing rate, punch pressure should be at 21–22 MPa and press descent speed should be at 24–26%.

In the graphs in Figure 3e,f, the interaction of the parameters blank holder pressure and punch pressure was shown. Blank holder pressure varied between 6 and 10 MPa and punch pressure varied between 18 and 22 MPa. From the graphs, it is seen that the earing rate drops to the lowest levels (approximately 4.0–4.5%) when blank holder pressure is around 9.5–10 MPa and punch pressure is in the range of 21.5–22 MPa. The appropriate combination of these two parameters is important for the homogeneity of the deep drawing process.

As a result, these response surface graphs obtained with the Box–Behnken design show that the parameters blank holder pressure, punch pressure, and press descent speed should be effectively controlled in the deep drawing process and that the earing rate can be minimized with appropriate combinations. These numerical analyses contribute to the improvement of the production process and make it possible to obtain more homogeneous and high-quality products.

**Figure 4:**
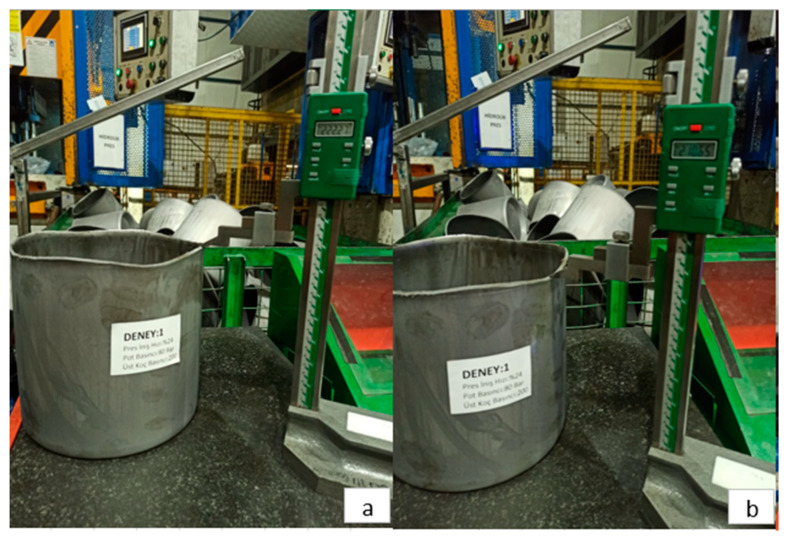
(**a**) Maximum elongation and (**b**) minimum elongation in Experiment 1.

Figure 4 presents images related to the deep drawing process with a press descent speed of 24%, a blank holder pressure of 8 MPa, and a punch pressure of 20 MPa. As a result of the deep drawing process, the maximum elongation measured is 222.27 mm, while the minimum elongation is 210.65 mm.

**Figure 5:**
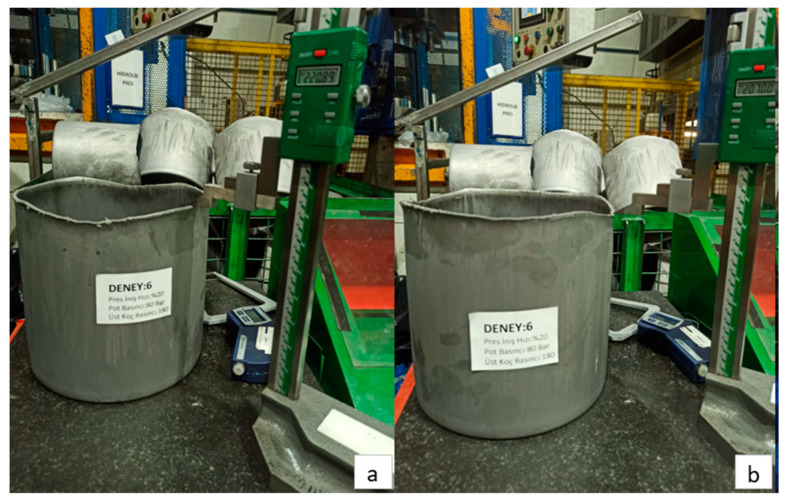
(**a**) Maximum elongation and (**b**) minimum elongation in Experiment 6.

Images related to the deep drawing process conducted with a press descent speed of 20%, a blank holder pressure of 8 MPa, and a punch pressure of 18 MPa are presented in Figure 5. As a result of this process, the maximum elongation measured is 220.89 mm, while the minimum elongation is determined to be 207.00 mm.

In the context of our study, it is essential to define the terms “Upper region” and “Lower region” as they pertain to the deep-drawn cylindrical sample. The upper region refers to the cylindrical section located 25 mm below the upper edge of the deep-drawn and trimmed sample (Figure 1c). The average measurement of three randomly selected points within this area is referred to as the “upper region final thickness.” Conversely, the lower region pertains to the area located 25 mm above the lower edge of the final deep-drawn sample (Figure 1c), where the average measurement of three randomly selected points is termed the “lower region final thickness”. Furthermore, the thinning ratios for all specimens have been calculated using the formula given in Table 5.

The thinning ratios obtained from the samples resulting from the experiments designed using the Box–Behnken design are presented in Table 5. The analysis of variance (ANOVA) related to the thinning ratios is provided in Table 6.

According to the ANOVA results presented in Table 6, the R^2^ and Adj-R^2^ values for Equation (3) are determined to be 0.970 and 0.904, respectively. These statistical indicators demonstrate that the model’s predictive capability is quite robust.

**Figure 6:**
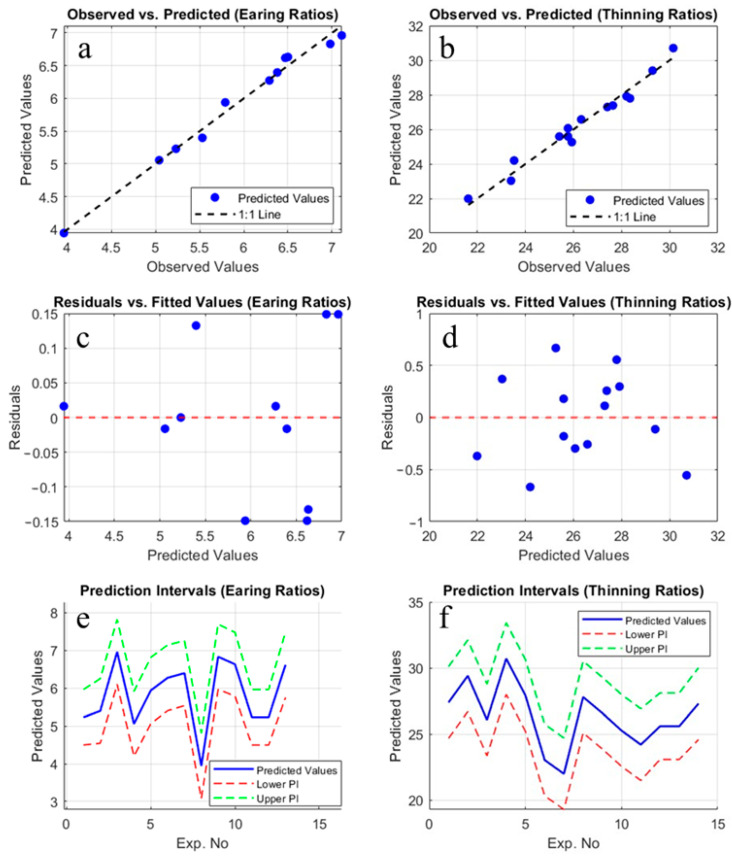
Model performance evaluation for earing and thinning ratios. (**a**) Observed vs. predicted values for earing ratios, demonstrating the model’s predictive accuracy. The dashed line represents the ideal 1:1 fit. (**b**) Observed vs. predicted values for thinning ratios, showing a strong correlation between observed and predicted values. (**c**) Plot of residuals vs. fitted values for earing ratios, which helps assess the assumption of homoscedasticity and the randomness of residuals. (**d**) Plot of residuals vs. fitted values for thinning ratios, providing insights into potential heteroscedasticity or systematic patterns in residuals. (**e**) Prediction intervals for earing ratios, where the blue solid line represents predicted values, and the red dashed and green dashed lines represent lower and upper prediction intervals, respectively. (**f**) Prediction intervals for thinning ratios, with the same representation as in (**e**), ensuring uncertainty quantification of the model’s predictions.

To enhance the accuracy and reliability of the model, the analyses conducted in this study were not limited to Minitab but were also re-executed using MATLAB 2024b software under the TU Delft license. During this additional validation process, the model was reconstructed and calculations were independently tested. The results obtained demonstrated consistency with the analyses performed using Minitab (Minitab 22.0, Minitab Inc., State College, PA, USA), thereby reinforcing the reliability of the model through verification via two different software platforms. This validation process strengthens the reproducibility of the model and the methodological robustness of the study, further supporting the validity of the findings. Figure 6 illustrates the model performance evaluation for earing and thinning ratios. The observed vs. predicted values (Figure 6a,b) highlight the model’s predictive accuracy and strong correlation. The plots of residuals vs. fitted values (Figure 6c,d) assess homoscedasticity and detect systematic residual patterns. Prediction intervals (Figure 6e,f) quantify uncertainty, with predicted values shown in blue and bounds in red and green.

**Figure 7:**
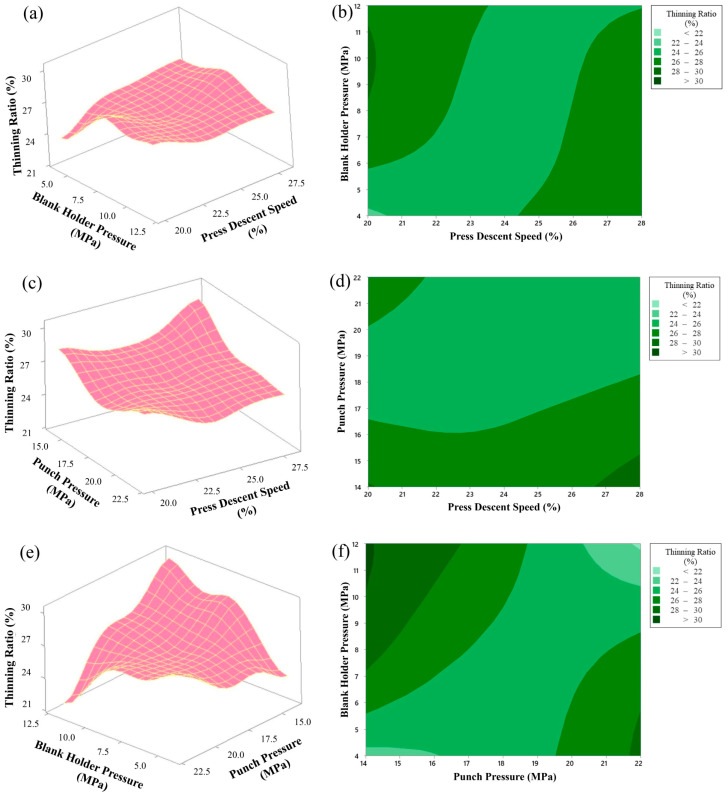
Surface and contour plots showing simultaneous effects of blank holder pressure, punch pressure and press descent speed on the thinning ratio, (**a**) 3D surface plot of thinning ratio as a function of blank holder pressure and press descent speed, (**b**) contour plot of thinning ratio as a function of blank holder pressure and press descent speed, (**c**) 3D surface plot of thinning ratio as a function of punch pressure and press descent speed, (**d**) contour plot of thinning ratio as a function of punch pressure and press descent speed, (**e**) 3D surface plot of thinning ratio as a function of blank holder pressure and punch pressure, (**f**) contour plot of thinning ratio as a function of blank holder pressure and punch pressure.

Response surface and contour graphs examining the effects of three main parameters (blank holder pressure, punch pressure and press descent speed) that affect the thinning ratio in the deep drawing process are given in Figure 7. These graphs, obtained using the Box–Behnken design, evaluate the effects of the parameters on the thinning ratio in detail.

In the graphs in Figure 7a,b, the effects of the variables blank holder pressure (MPa) and press descent speed (%) on the thinning ratio are analyzed. Blank holder pressure varies between 4 and 12 MPa, and press descent speed varies between 20% and 28%. In the graphs, it is seen that the thinning ratio reaches minimum values (<22%) when blank holder pressure is in the range of 4 MPa and press descent speed is at approximately 20%. This combination can be considered as an important control point in order to reduce the risk of heterogeneous thinning in the material and maintain product quality.

The effects of the parameters punch pressure (MPa) and press descent speed (%) on the thinning rate are shown. Punch pressure varies between 14 MPa and 22 MPa, while press descent speed is again between 20% and 28%, as seen in Figure 7c,d. It is understood that the thinning rate is lower (approximately 24–26%) when punch pressure is at 18–20 MPa and press descent speed is at 24–26%. Low thinning rate can contribute to more homogeneous shaping of the material and prevention of undesirable deformations.

As seen in Figure 7e,f, the interaction of the parameters blank holder pressure (MPa) and punch pressure (MPa) is examined. Blank holder pressure varies between 4 and 12 MPa, while punch pressure varies between 14 and 22 MPa. It is observed that the thinning ratio remains at minimum levels (approximately 22–24%) in the ranges of blank holder pressure 11–12 MPa and punch pressure 21–22 MPa. This is an important result for both the proper molding process and the preservation of the quality of the material.

As a result, these response surface graphics obtained with the Box–Behnken design reveal that the parameters blank holder pressure, punch pressure and press descent speed should be effectively controlled in order to minimize the thinning ratio in the deep drawing process. These numerical analyses provide significant contributions to the optimization of the production process and the increase in product quality by keeping the thinning ratio under control.

**Figure 8:**
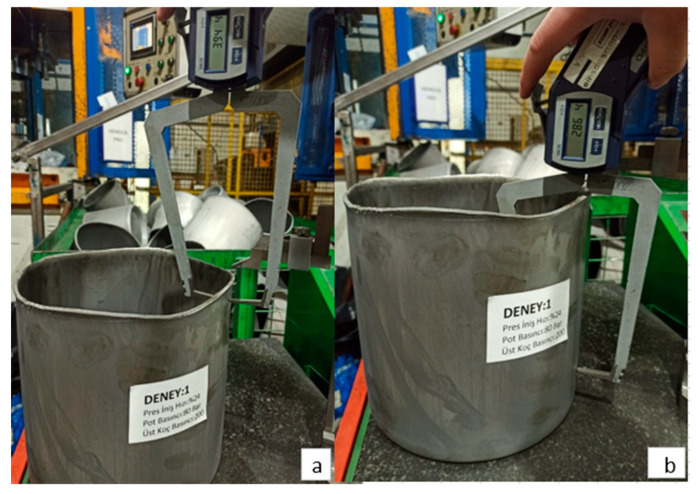
For Experiment 1: (**a**) upper region thinning amount, (**b**) lower region thinning amount.

Figure 8 shows images of the deep drawing process with a press descent speed of 24%, a blank holder pressure of 8 MPa and a punch pressure of 20 MPa. As a result of the deep drawing process, the upper region thinning amount was measured as 3.94 mm and the lower region thinning amount was measured as 2.86 mm.

**Figure 9:**
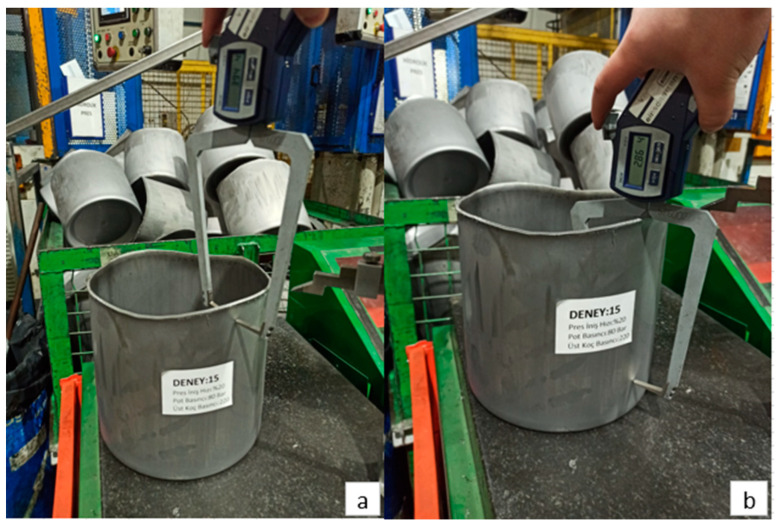
(**a**) Upper region thinning amount and (**b**) lower region thinning amount for Exp. 15.

In Figure 9, the post-process images of the deep drawing process performed according to the process parameters of press descent speed, blank holder pressure and punch pressure set at 20%, 8 MPa and 22 MPa, respectively, are shown. As a result of the deep drawing process, the thinning amount in the upper region was determined as 3.94 mm and the thinning amount in the lower region was determined as 2.86 mm.

### 3.2. Optimization Results

In this study, the PSO algorithm was selected due to its robustness in handling complex, multi-dimensional optimization problems. The primary goal was to minimize the defects in the deep drawing process, particularly focusing on reducing material thinning and earing, which are common issues in sheet metal forming. The PSO method works by simulating a swarm of particles that move within the solution space, adjusting their positions based on their own experience and the experience of neighboring particles to converge toward an optimal solution.

The optimization algorithm was employed to minimize two target functions: the earing ratio (y_1_) and the thinning ratio (y_2_). Both of these metrics were optimized using Particle Swarm Optimization (PSO), as detailed in the methodology section. Based on the algorithm flow diagram presented in Figure 2, the MATLAB code was written to guide the input parameters towards achieving the global minimum of the target functions. For the earing ratio, the optimal input parameter values for x_1_, x_2_, and x_3_ were found to be 27.38, 10, and 22, respectively. These values were found to minimize the formation of ears in the drawn part, not only leading to a more uniform product but also reducing the post-processing cost. Similarly, for the thinning ratio, the PSO algorithm identified the optimal values of x_1_ = 26.55, x_2_ = 10, and x_3_ = 22, which helped in maintaining a consistent wall thickness, minimizing the risk of failure during the deep drawing process. These findings are crucial for improving the overall quality and efficiency of the forming process. The parameter values obtained through PSO have successfully achieved optimization in minimizing both the earing and thinning ratios. These findings provide a significant foundation for enhancing the quality of deep drawing processes and optimizing production methods, while also carrying the potential to improve efficiency in industrial applications.

**Figure 10:**
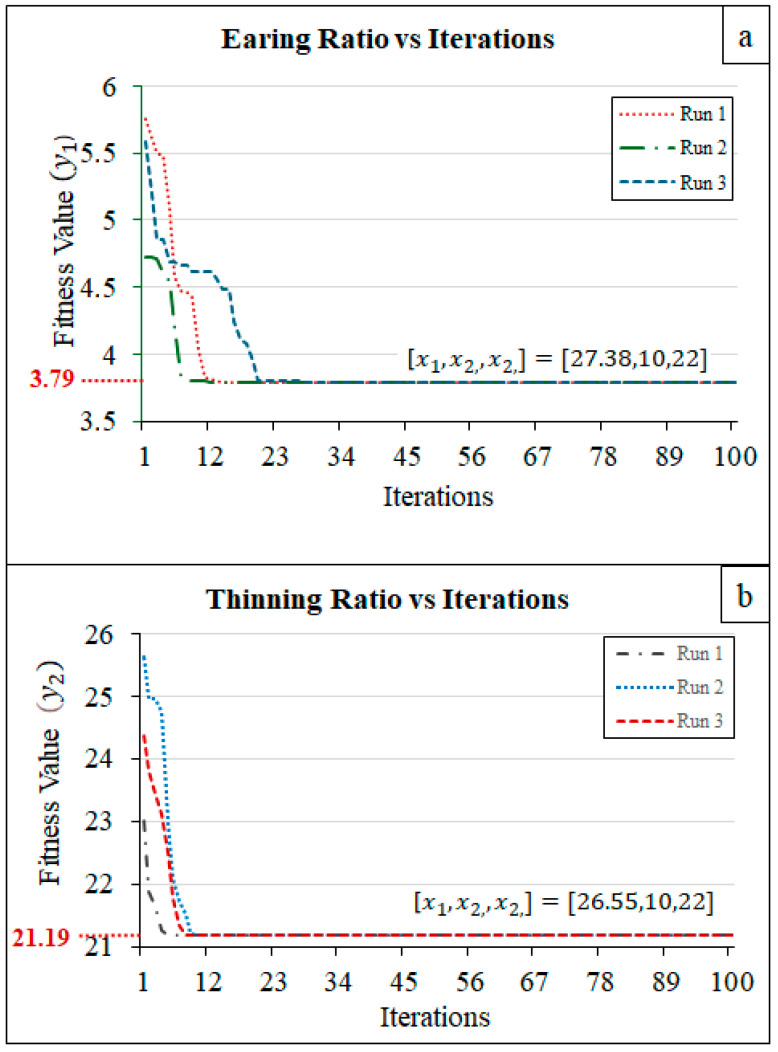
Iterative optimization results of (**a**) earing ratio and (**b**) thinning ratio.

The earing ratio, a metric indicative of the process’s quality or efficiency, exhibited a general downward trajectory across all three runs as the iteration count increased. This trend suggests convergence towards an optimal solution. However, the rate of this decline varied among the runs. Run 1 demonstrated the most rapid decrease, while Run 3 exhibited the slowest. The final earing ratio attained is 3.79 for all runs (Figure 10a).

Similarly, the thinning ratio, a parameter related to the inhomogeneity of the wall thickness, also displayed a decreasing tendency with increasing iterations. This indicates a progressive reduction in thinning throughout the optimization process. The variability in the thinning ratio’s behavior across the runs was more pronounced than that of the earing ratio. Run 1 and Run 2 experienced steeper declines, whereas Run 3 demonstrated a more gradual reduction. The final thinning ratio for all runs reaches 21.19, as shown in Figure 10b.

Overall, the results indicate that both the earing ratio and thinning ratio converged towards specific values as the optimization process progressed. The disparities in convergence rates and overall behavior among the runs underscore the sensitivity of the optimization process to initial conditions or parameters. While the graphs alone do not explicitly reveal the relationship between the earing ratio and thinning ratio, further analysis or contextual understanding is necessary to elucidate this connection.

The optimization of the earing ratio and uniform thickness in the deep drawing process is a critical area of study within manufacturing and materials engineering. Our results indicate that the earing ratio, which is a measure of the variation in height of the drawn part, can be significantly influenced by processing parameters. Specifically, we observed that an increase in the blank holder force leads to a reduction in the earing ratio. This finding aligns with previous studies who reported similar trends in their investigations [17,20,21]. An increase in blank holder pressure has been shown to significantly reduce the earing ratio during the deep drawing process. This correlation arises because a higher blank holder pressure effectively controls the material flow, minimizing the likelihood of uneven deformation that leads to earing. Research indicates that as it increases, the material is better constrained, which helps maintain a more uniform thickness across the drawn part, thereby reducing earing. The reduction in earing can be attributed to the enhanced control over material flow, which minimizes the formation of irregularities during the drawing process [17,22,23].

Moreover, the uniformity of thickness throughout the drawn part is paramount for ensuring the structural integrity and performance of the final product. Our optimization studies revealed that maintaining the maximum blank holder pressure as well as the maximum punch pressure, within the technical limits of the hydraulic press, under an intermediate value of press descent speed can significantly enhance thickness uniformity. This observation is consistent with the previous works [24,25].

Despite the advancements presented in this study, several limitations warrant discussion. The experimental conditions were confined to specific materials and geometries, which may not be universally applicable. Future research should aim to explore a broader range of materials and complex geometries to validate and extend the findings of this study. Additionally, the long-term effects of the optimized parameters on the mechanical properties of the drawn parts remain to be investigated.

## 4. Conclusions

This study focuses on determining the optimum values of input parameters, such as press descent speed and blank holder pressure, in the deep drawing process of DD13-grade sheets using a 250-ton hydraulic press in terms of earing and thinning ratio parameters. Achieving uniform material thickness and minimizing earing after the plastic deformation process is crucial for the deep drawing process.

Upon examining the results of this study, it was found that the minimum earing ratio was achieved at a press descent speed of 27.38%, a blank holder pressure of 10 MPa, and a punch pressure of 22 MPa. Additionally, the thickness closest to uniform was obtained with a press descent speed of 26.55%, a blank holder pressure of 10 MPa, and a punch pressure of 22 MPa. As observed, the same input combination is not sufficient to achieve the optimum values for different output parameters. In this context, it would be appropriate to determine the process parameters separately for each desired target (earing ratio, thinning ratio).

Based on the knowledge gained from pilot experiments, regression models were developed for the earing and thinning ratio response parameters using the Box–Behnken design matrix, yielding R^2^ values of approximately 0.98 and 0.97, respectively. These indicators demonstrate that the models possess strong predictive capabilities. Using the regression models obtained in this study, it is possible to predict the earing and thinning ratios without the need for further experimentation based on the input parameters. In this regard, the results of the study provide valuable contributions in reducing the need for labor-intensive processes in industrial contexts regarding time and cost. The proposed methodological steps in this study can be applied to a wide range of different manufacturing processes, making this method a useful tool.

## Figures and Tables

**Table 1:** Chemical composition of DD13 steel (%).

Experiment Material	Chemical Composition (%)				
**DD13**	C	Mn	S	P	Fe
	0.08	0.46	0.03	0.03	Balance

**Table 2:** Deep drawing parameters and levels for BBD.

Symbols	Parameters	Levels		
		−1	0	1
$A(x_{1}$)	Press Descent Speed (%)	20%	24%	28%
$B(x_{2}$)	Blank Holder Pressure (MPa)	6	8	10
$C(x_{3}$)	Punch Pressure (MPa)	18	20	22

**Table 3:** BBD earing ratio experimental results.

Exp. No	Press Descent Speed (%)	Blank Holder Pressure (MPa)	Punch Pressure (MPa)	Min. Height (mm)	Max. Height (mm)	Earing Ratio ** (%)
1	24	8	20	210.65	222.27	5.23
2	20	10	20	210.96	223.30	5.53
3	28	8	18	207.75	223.64	7.11
4	28	8	22	212.53	223.80	5.04
5	24	10	18	212.20	225.25	5.79
6	20	8	18	207.00	220.89	6.29
7	24	6	18	208.20	222.39	6.38
8	24	10	22	211.79	220.52	3.96
9	24	6	22	209.23	224.94	6.98
10	28	6	20	208.01	222.48	6.50
11 *	28	10	20	210.31	224.89	6.48
12 *	20	6	20	210.24	221.73	5.18
13	24	8	20	210.72	222.35	5.23
14	24	8	20	210.68	222.31	5.23
15	20	8	22	208.60	223.04	6.47
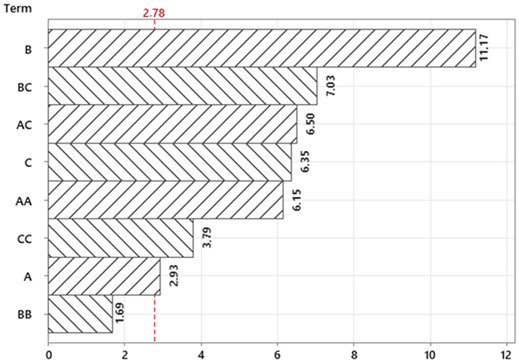						

* Outliers. ** Earing Ratio = $\frac{\left(Maximum\;Height-Minimum\;Height\right)}{Maximum\;Height}\times 100.$

**Table 4:** ANOVA results for the BBD earing ratio experiment.

Source	DF	Sum of Squares	Mean Squares	F-Value	*p*-Value
Model	8	9.52599	1.19075	39.64	0.002
Linear	3	2.00497	0.66832	22.25	0.006
x1	1	1.28264	1.28264	42.70	0.003
x2	1	0.50432	0.50432	16.79	0.015
x3	1	0.19085	0.19085	6.35	0.065
Quadratic Terms	3	1.78554	0.59518	19.81	0.007
x1 * x1	1	1.13777	1.13777	37.87	0.004
x2 * x2	1	0.08599	0.08599	2.86	0.166
x3 * x3	1	0.43331	0.43331	14.42	0.019
Interactive Terms	2	2.75784	1.37892	45.9	0.002
x1 * x3	1	1.27171	1.27171	42.33	0.003
x2 * x3	1	1.48613	1.48613	49.47	0.002
Error	4	0.12017	0.03004		
Lack of Fit	2	0.12016	0.06008	17681.1	
Pure Error	2	0.00001	0		
Total	12	9.64616			

**Table 5:** BBD thinning ratio experimental results.

Exp. No	Press Descent Speed (%)	Blank Holder Pressure (MPa)	Punch Pressure (MPa)	Upper Region Final Thickness	Lower Region Final Thickness	Thinning Ratio ** (%) (Ratio of the Thickness Difference in the Upper and Lower Regions)
1 *	24	8	20	2.86	2.86	0.00
2	20	10	20	3.98	2.88	27.64
3	28	8	18	3.96	2.80	29.29
4	28	8	22	3.88	2.88	25.77
5	24	10	18	3.98	2.78	30.15
6	20	8	18	3.90	2.80	28.21
7	24	6	18	3.76	2.88	23.40
8	24	10	22	3.70	2.90	21.62
9	24	6	22	3.88	2.78	28.35
10	28	6	20	3.80	2.80	26.32
11	28	10	20	3.78	2.80	25.93
12	20	6	20	3.74	2.86	23.53
13	24	8	20	3.70	2.76	25.41
14	24	8	20	3.88	2.88	25.77
15	20	8	22	3.94	2.86	27.41
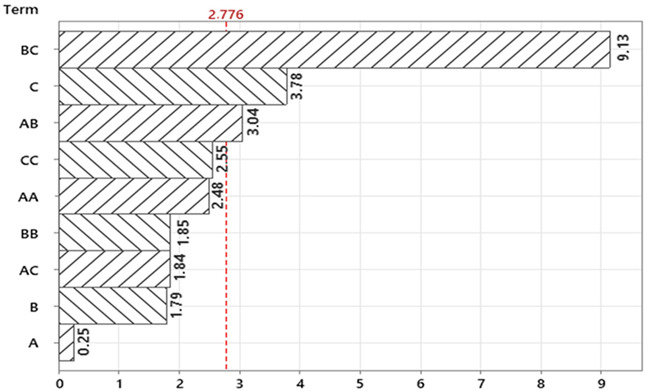						

* Outliers. ** Thinning Ratio = $\frac{\left(Upper\;Region\;Final\;Thickness-Lower\;Region\;Final\;Thickness\right)}{Upper\;Region\;Final\;Thickness}\times 100.$

**Table 6:** ANOVA results for the BBD thinning ratio experiment.

Source	DF	Sum of Squares	Mean Squares	F-Value	*p*-Value
Model	9	71.8876	7.9875	14.68	0.010
Linear	3	42.1878	14.0626	25.84	0.004
X1	1	4.6472	4.6472	8.54	0.043
X2	1	32.7669	32.7669	60.22	0.001
X3	1	0.2671	0.2671	0.49	0.522
Quadratic Terms	3	10.0002	3.3334	6.13	0.056
X1 * X1	1	3.3681	3.3681	6.19	0.068
X2 * X2	1	1.8624	1.8624	3.42	0.138
X3 * X3	1	3.5643	3.5643	6.55	0.063
Interaction Terms	3	52.3135	17.4378	32.05	0.003
X1 * X2	1	5.0594	5.0594	9.30	0.038
X1 * X3	1	1.8575	1.8575	3.41	0.138
X2 * X3	1	45.3965	45.3965	83.42	0.001
Error	4	2.1766	0.5442		
Lack of Fit	3	2.1090	0.7030	10.39	0.223
Pure error	1	0.0676	0.0676		
Total	13	74.0643			

## Data Availability

This article has no additional data. All data are included in this study.
